# Acousto-microfluidics for screening of ssDNA aptamer

**DOI:** 10.1038/srep27121

**Published:** 2016-06-08

**Authors:** Jee-Woong Park, Su Jin Lee, Shuo Ren, Sangwook Lee, Soyoun Kim, Thomas Laurell

**Affiliations:** 1 Department of Biomedical Engineering, Lund University, Lund, Sweden; 2 Department Biomedical Engineering, Dongguk University, Seoul, Republic of Korea; 3 Department of Chemistry, The University of Tokyo, Tokyo, Japan

## Abstract

We demonstrate a new screening method for obtaining a prostate-specific antigen (PSA) binding aptamer based on an acoustofluidic separation (acoustophoreis) technique. Since acoustophoresis provides simultaneous washing and separation in a continuous flow mode, we efficiently obtained a PSA binding aptamer that shows high affinity without any additional washing step, which is necessary in other screening methods. In addition, next-generation sequencing (NGS) was applied to accelerate the identification of the screened ssDNA pool, improving the selecting process of the aptamer candidate based on the frequency ranking of the sequences. After the 8^th^ round of the acoustophoretic systematic evolution of ligands by exponential enrichment (SELEX) and following sequence analysis with NGS, 7 PSA binding ssDNA aptamer-candidates were obtained and characterized with surface plasmon resonance (SPR) for affinity and specificity. As a result of the new SELEX method with PSA as the model target protein, the best PSA binding aptamer showed specific binding to PSA with a dissociation constant (*K*_*d*_) of 0.7 nM.

Aptamer refers to short single-stranded nucleic acid that folds into a three-dimensional structure for efficient binding to their specific target with a high affinity^1^,^2^,^3^. Aptamers show high affinity for a wide variety of molecules ranging from small molecules^4^ or even metal ions^5^ to complex molecules such as proteins^6^, viruses^7^ and whole cells^8^. Because aptamers possess high affinity to their specific targets and offer many advantages in terms of method of development and production cost, aptamers have been applied in various fields of research including therapeutics^9^, biosensors^10^,^11^,^12^, purification systems^13^,^14^, bio-imaging systems^15^,^16^,^17^, etc. For the screening of aptamers, systematic evolution of ligands by exponential enrichment (SELEX), in which binding, separation, and amplification of nucleic acid fragment can be altered to accommodate to the various needs, has been used. Since the first report in 1990^1^,^3^, significant technological progress has been made to improve previous methodologies for efficient separation of target-bound ssDNA from unbound ssDNA^18^,^19^,^20^,^21^.

In SELEX, the most critical step is to design a system for efficient separation of the target-bound ssDNA from unspecific DNA molecules not bound to the target. A highly efficient separation can increase the affinity of the final aptamer candidates^22^ and reduce cost, time and effort by lowering the number of rounds of selection. Even though various matrixes for the separation are applied to aptamer selection processes^23^,^24^,^25^, method developments for reducing cost, labor and experimental times are still required. Hence, in order to address these issues, many microfluidic based techniques have been applied for enhancing efficiency of separation, including capillary electrophoresis (CE)^26^,^27^,^28^, sol-gel technique^29^, and magnetic-microfluidics^30^,^31^.

In this study, we describe a new microfluidic SELEX method based on acoustophoresis, which offers a rapid and continuous flow based process for the simultaneous separation and washing in order to enable the efficient enrichment of aptamer candidates from ssDNA libraries. No additional washing steps are needed which commonly is a requisite for other SELEX methods. By removing additional washing steps, the possibility of target loss is minimized as well as time and labor.

In the principle of continuous flow based acoustophoresis, a standing wave field induces particle movement depending on its acoustic properties such as diameter, density or compressibility^32^. In the acoustophoretic channel with sheath flow, the particles are firstly laminated to the side of the channel by a central sheath flow of clean buffer and then merged into clean buffer due to the acoustic radiation force, based on their acoustophoretic mobility. Because the migration of particles is strongly governed by the size, which is related to the second power of the radius, small particles and molecular species are moderately or not at all influenced by the acoustic standing wave force in comparison to rigid particles in the 3–10 μm range^33^. Since acoustophoresis offers an efficient continuous flow based separation mode of operation with reasonable throughput, acoustophoresis in microfluidic systems has been studied for various types of samples including blood^34^, raw milk^35^ and circulating tumor cells^36^.

We speculated that this separation technique could be advantageous for the screening of ssDNA aptamer since efficient washing and separation is the most critical process in screening of aptamers. This speculation was supported by previous studies where acoustophoresis in a continuous flow format was applied to an efficient separation of lymphocytes^37^, a purification of phosphopeptides^38^ or a selection of bacteriophage^39^ with functionalized micro beads. In the same manner, in order to screen the ssDNA aptamer, microbeads modified with a target molecule were in this study initially incubated with an ssDNA library and then injected into the acoustophoretic channel for simultaneous separation and washing. As the sample mixture entered the acoustic standing wave field, the micro-particles with target bound DNA-fragments migrated across the central buffer interface and exited the system through the central outlet, whereas the unbound proteins and ssDNAs remained in the original buffer stream along the side walls and were removed through the side outlets. The migration of beads by the acoustic wave force field from the side wall to the center of the clean buffer, enables simultaneous washing and separation in the continuous flow.

Next-generation sequencing (NGS) is a high throughput sequencing technique which enables the collection of large amounts of sequence data in relatively short periods of time^40^. The NGS technique has been applied in various fields such as medical research^41^ and ligand discovery research^42^. In addition, NGS enables the analysis of the high complexity of the enriched pool after performing several selection rounds of aptamer screening^43^. For those reasons, we combined NGS technique also in the new acoustophoretic SELEX method to improve efficiency and speed of the sequence analysis significantly.

In terms of the model target protein, prostate-specific antigen (PSA), which is a well-known biomarker of prostate cancer^44^,^45^ was selected for its importance in early diagnosis of prostate diseases. Because PSA screening tests are reported to have a significant correlation with 20% reduction in cancer mortality, requirements of screening highly accurate molecular probes are increasing to improve the sensitivity of PSA screening test and reduce false positive diagnosis. Ultimately a more specific assay than solely the PSA level as an indicator of prostate cancer is sought and current developments aim at providing multiplex assays where PSA in combination with other disease related biomarkers gives an improved diagnostic value.

In this study, we demonstrate a new method for obtaining highly PSA specific aptamers assisted by continuous flow mode acoustophoresis, which encompasses a simultaneous washing and separation method without additional washing steps in combination with NGS for efficient analysis of the obtained DNA pool.

## Result

### Acoustophoresis based SELEX

As a preliminary test for recovery of beads and separation efficiency, beads suspended in PBS (0.3 mg/ml) containing Evans blue (153.8 μg/ml) were injected into the acoustophoresis separation system under the same conditions as when performing the SELEX procedure (4.93 MHz for pre-focusing and 1.99 MHz for main focusing with a flow rate of 500 μl/min).

The recovery of washed beads were measured by a Coulter counter (Multisizer III; Beckman Coulter, Brea, CA, USA) and the bead recovery turned out to be 99.2% (the ratio of collected beads to inlet beads) and washing efficiency of Evans blue was 99.0% (intensity ratio of central outlet to side outlet). Similar to the model contaminant, Evans blue, small molecules are not affected by the primary acoustic radiation force, hence the unbound ssDNA molecules and counter-proteins can be discarded through the side outlets. On the other hand, the affinity microbeads on which ssDNA is bound are driven to the center of the channel across the buffer interface and can be collected from the central outlet.

**Figure 1:**
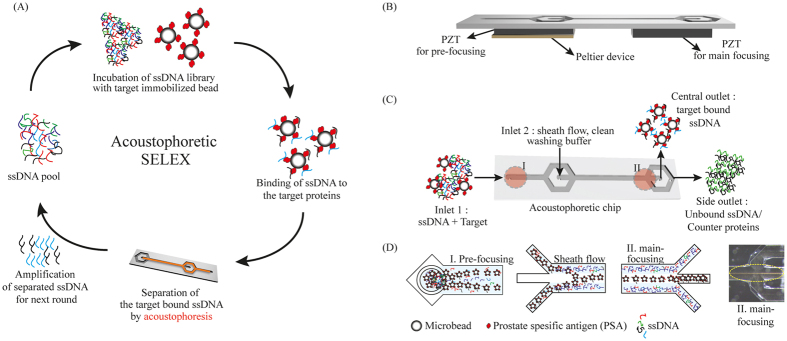
The schematic overview of the acoustophoretic device and SELEX. (**A**) The schematic flow of acoustophoretic SELEX. The ssDNA pool is incubated with main targets immobilized on the micro beads. Then the beads were simultaneously washed and separated from unbound ssDNA and counter targets by acoustophoresis. The target-bound DNA on the beads are collected and amplified by PCR for next round. (**B**) The composition of the acoustophoretic setup. The piezo electric transducers and Peltier device were attached to the acoustophoretic chip. (**C**) Flow of samples in acoustophoretic chip. Red circle indicates the location of each acoustophoretic process. The Roman letter I and II depicts the pre-focusing and the main focusing. (**D**) Detailed illustration of each process in the chip. In the pre-focusing area, two dimensionally focused streams are aligned (I). Target bound ssDNA on the beads going into the clean washing buffer stream while the unbound ssDNA remained along the channel sidewalls due to the difference of acoustic properties (II). Washed ssDNA on the beads are separated and collected through the central outlet while unbound ssDNA is discarded through the side outlet. The dashed line in the insert microscopic image shows the focused beads on which ssDNA bound to the target.

**Figure 2:**
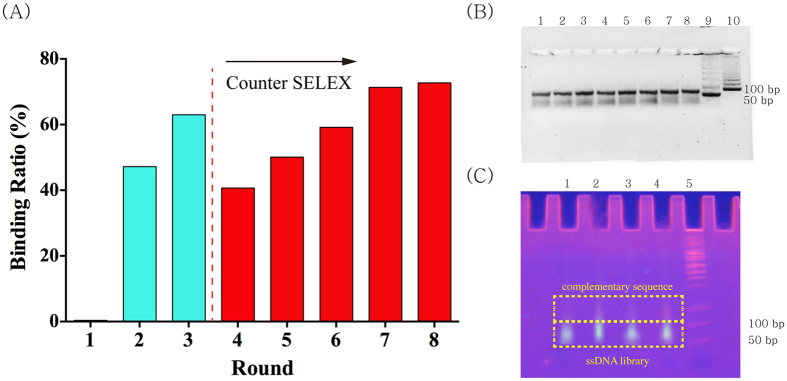
(**A**) The progress of the acoustophoretic SELEX. Binding ratio was ratio of the amount of bound ssDNA to the amount of input ssDNA. From the 4^th^ round, the counter SELEX was performed to increase the specificity. After the 8^th^ round, as the binding ratio reached to a saturated status, the ssDNA pool was analyzed by NGS. (**B**) Agarose gel analysis of PCR product. Lane 1 ~ 8: PCR product, Lane 9:50 base pair ladder, lane 10:100 bp ladder (**C**) The isolation of ssDNA fraction from PCR product with a polyacrylamide gel electrophoresis. Lane 1 ~ 4: PCR product, Lane 5:50 base pair ladder. Due to the T-tail of the reverse primer, mobility of the complementary sequence to the ssDNA library is slower than ssDNA library.

As a result of SELEX, the PSA binding aptamers were successfully selected from the random ssDNA library consisting of 1.2 × 10^14^ different sequence variants with acoustophoretic setup (Fig. 1). The SELEX process was subsequently repeated until the recovery ratio (amount of bound ssDNA/amount of input DNA × 100%) reached plateau at approximately 70% after 8^th^ round (Fig. 2). After the pool reached the saturation status, the pool of remaining ssDNA was analyzed by NGS to identify the obtained sequences.

### Next-generation sequencing

**Figure 3:**
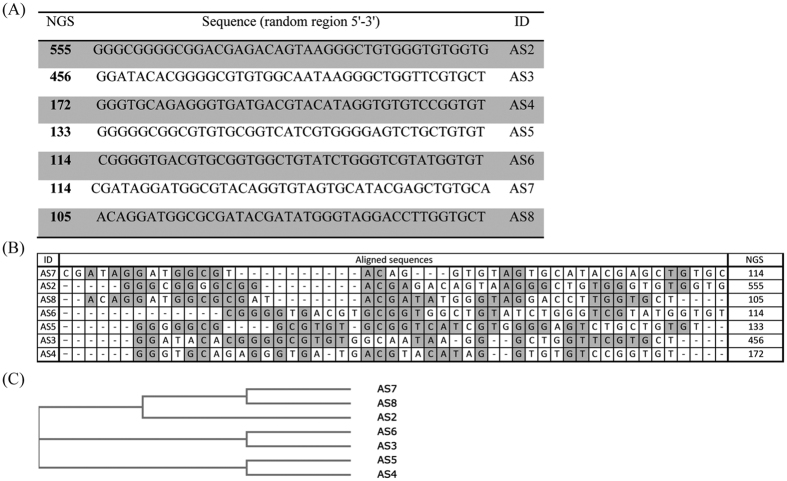
PSA binding aptamer candidates obtained from the acoustophoretic SELEX (**A**) The sequences of PSA binding aptamers without primer region. (**B**) The aligned sequences of the candidates, The NGS numbers indicate the individual sequence’s frequency of occurrence. The repeated sequences are shaded in gray. (**C**) Phylogenetic tree that shows the homology of each sequences.

After 8^th^ round of acoustophoretic SELEX, PSA binding ssDNA sequences were analyzed by NGS. As a result of NGS analysis, we obtained 7 sequences showing the highest abundance among approximately 1.7 × 10^4^ raw sequences of data we got after 8^th^ round of acoutophoresis SELEX (Fig. 3). The similarity among the aptamer candidates was analyzed through homology tree and sequence alignment^46^ in Fig. 3.

### Characterization of aptamer candidates by BIAcore

**Figure 4:**
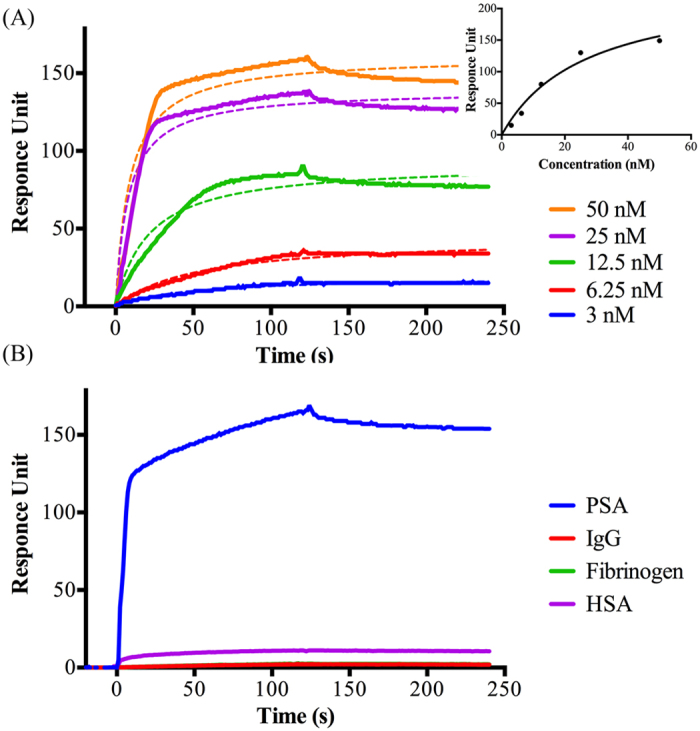
Affinity and specificity of the PSA aptamer AS2 analyzed by the Biacore SPR system. (**A**) The various concentrations of AS2 show the binding reaction and the calculated dissociation constant is 0.7 nM. Dashed lines show the fitted curve of non-linear regression. Insert shows the fitting result of each binding curve with Michaelis-Menten kinetics. (**B**) The specificity of AS2, which bind to PSA and does not bind to the counter targets (IgG, fibrinogen and HSA). The AS2 (100 nM) is injected to the Biacore chip treated with each protein of 10 μg/ml.

The 7 selected sequences were characterized by measuring the equilibrium dissociation constants (*K*_*d*_) and binding specificity with Biacore SPR instrumentation^47^. After analyzing the 7 candidates, AS2, which had the highest NGS score, turned out to have the strongest affinity with highest specificity to PSA (Fig. 4) with a *K*_*d*_ value calculated to 0.7 nM. The binding of AS2 to PSA was plotted in a Michaelis-Menten curve with R^2^ of 0.96. The dissociation constant value of 0.7 nM is the equivalent value to the strongest reported PSA binding antibody^48^ (Table 1). Regarding the specificity, AS2 showed specific binding to PSA when the same amount of AS2 was treated to three protein coated flow cells. It is shown that AS2 binds to PSA and not to any of the counter proteins (Fig. 4). In Fig. 4(B), the binding curve of AS2 to the fibrinogen is obscured by the curve of IgG.

## Discussion

The acoustophoretic separation (acoustophoresis) method has been reported as a simple, rapid and cost-effective separation technique^49^,^50^. Continuous flow based acoustophoresis utilizes a standing wave field that moves the rigid particles (having a positive acoustic contrast factor) into the acoustic pressure node which is surrounded by a clean buffer stream at the center of the channel while the original sample buffer remain along the side of the channel^51^ (Fig. 1).

The acoustic microfluidic chip used in this study consists of two inlets and two outlets (Fig. 1). The first inlet is for the sample and the second inlet is for the sheath flow of the clean washing buffer. Regarding the outlets, the central outlet is intended for the washed and separated beads with bound ssDNA that have been transported by the acoustic standing wave force field, and the side outlet collects unbound ssDNA molecules and counter protein.

In this acoustic chip, the beads will flow through two different acoustic regions, which is the pre-focusing region of width and height dimensions 300 μm by 150 μm and the main-focusing region of dimensions 375 μm by 150 μm (Fig. 1). Firstly, in the pre-focusing region, all beads are located in the same flow position of the parabolic flow with two dimensionally focused streams^36^ by inducing 5 MHz of the acoustic standing wave. Since the 5 MHz standing wave matches half a wavelength across the height of the channel (150 μm) and one wavelength across the width of the channel (300 μm), beads can be aligned into 2 lines close to the side wall with two dimensional focusing.

In the subsequent main focusing region (375 μm wide), actuated at 2 MHz in a half wavelength resonance, the beads are separated from unbound ssDNA and simultaneously washed. The two lines of beads laminated along the side wall migrate into the clean buffer and merge into one stream line of beads in the center of the clean buffer flow completing the separation of beads to the central outlet and washing the unbound ssDNA to the side outlet when the acoustic force field is actuated. Because of the laminar flow, beads can be maintained in a two-dimensional stream in the main focusing channel as well as in the pre-focusing channel. The most advantageous process of the continuous flow based acoustophoretic SELEX comparing to another microfluidic-based SELEX is the simultaneous separation of target beads and washing of the unbound ssDNA due to the sheath flow and efficient acoustophoretic lateral migration of the selection beads across the sheath flow boundary. This advantage enables the elimination of additional washing steps at the same time as the separation efficiency of the bound to unbound target typically corresponds to a log 3 ratio^38^. Due to the elimination of additional washing steps, not only required time and labor but also the possible loss of target DNA during further washing steps is minimized.

Moreover, the continuous flow based acoustophoresis has enabled multiplex separation process^52^ and recirculation of separated bead samples with a gated collection process^53^. Therefore, although not yet realized in an aptamer selection process, this continuous flow based separation further enables the possibility of sequentially switching subsequent ssDNA samples for next round through the system.

Supported by these benefits, the continuous flow based acoustophoretic SELEX method was developed for screening aptamers with simultaneous separation and washing of the unbound ssDNA molecules (Fig. 1).

Briefly, the ssDNA library was incubated with PSA immobilized microbeads and subject to acoustophoretic washing of unbound ssDNA. After the 3^rd^ round of SELEX, the ssDNA pool was incubated with target immobilized microbeads dissolved in a mixture of counter targets, which exist most abundantly in blood, including HSA, IgG and fibrinogen for the higher specificity. After injecting this mixed solution into the acoustophoresis system, not only unbound ssDNA, but counter targets and counter targets-bound ssDNA were also eliminated. By repeating this washing procedure until the amount of bound ssDNA was saturated at 8^th^ round (Fig. 2), the screened PSA binding aptamer candidates were characterized.

After the acoustophoretic SELEX, sequences of the final DNA pool were analyzed by NGS (Fig. 3). The conventional sequencing method, which consists of cloning based on random picking of colonies and Sanger sequencing, gives information of selected colonies only. The whole process takes more than three days, providing limited sequencing information and difficulty in finding identical sequences. On the other hand, NGS enables sequencing of millions of DNA and provides whole sequence information of an ssDNA pool in a single run. Moreover, NGS provides high throughput ranking of data because all the raw sequences are counted and ranked based on sequence-frequencies^43^,^54^. The most highly ranked sequences can be selected as the aptamer candidates for further tests.

The 7 sequences obtained by NGS were evaluated for affinity and specificity with an SPR binding assay (Fig. 4). In order to measure *K*_*d*_ values via a standard SPR binding assay^55^, the main target PSA was immobilized onto the surface of a CM5 sensor chip. For the specificity test of the aptamer candidates, each of the counter proteins (HSA, IgG, and fibrinogen) were also immobilized onto the respective CM5 chips. Since the CM5 chip is coated by carboxymethylated dextran, it provides a hydrophilic environment and facilitates the immobilization of amino functionalized molecules.

After immobilization of PSA onto the CM5 chip, various concentrations of label free aptamer candidates, between 3 and 50 nM, dissolved in binding buffer were injected. Before the injection the aptamer dilutions were denatured at 90 °C for 10 min, followed by cooling to 4 °C for 15 min, and then incubated at 25 °C by using the thermo cycler to obtain a stable secondary ssDNA structure. The response units of aptamers bound to PSA or other counter proteins were determined and fitted with the BIAevaluation software using a 1:1 Langmuir model of binding. Each SPR curve was plotted for kinetic analysis with equation of 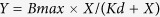 and the R^2^ value for goodness of fitting is calculated to be 0.96. The kinetic dissociation constant (*K*_*d*_) was determined by BIAcore evaluation software as well.

Due to the efficient separation and washing based on acoustophoresis, the *K*_*d*_ value of PSA specific aptamer AS2 turned out to be 0.7 nM, which is close to the *K*_*d*_ value, 0.3 nM of the antibody^48^ (Table 1).

As a conclusion, a new microfluidic SELEX method based on microchip acoustophoresis was developed, which shows a stringent separation ability known as the most critical factor for the screening of aptamer. This acoustophoretic SELEX enables simultaneous washing and separation in a continuous flow manner. With this new SELEX method in combination with NGS, a PSA specific ssDNA aptamer (AS2) was successfully isolated. The affinity specific feature of the obtained sequence was characterized using the BIAcore SPR platform for calculation of affinity constant to PSA and specificity against its counter targets. The new PSA aptamer AS2 showed a *K*_*d*_ of 0.7 nM and did not show any non-specific binding to the counter targets. This new PSA binding aptamer AS2 can be applied not only to aptamer research, but has potential to be applied in diagnosis systems for detection of prostate cancer.

## Methods

### Reagents

Prostate specific antigen (PSA), human serum albumin (HSA), IgG, fibrinogen (Fib), N-ethyl-N′-(dimethylaminopropyl) carbodiimide (EDC), N-Hydroxysulfosuccinimide sodium salt (NHS), and ethanolamine were purchased from Sigma-Aldrich (St Louis, USA). All other chemicals were of analytical grade and used without further treatment or purification. Double-distilled water (Millipore) was used in all experiments.

The ssDNA library comprised a randomized region of 40 (N40) nucleotides flanked by two constant regions allowing primer annealing and PCR amplification (Library: 5′-CGTACGGAATTCGCTAGC-N40-GGATCCGAGCTCCACGTG-3′, Forward primer sequences: 5′-fluorescein-CGTACGGAATTCGCTAGC-3′ and reverse primer sequence: 5′-T20-CACGTGGAGCTCGGATCC-3′).

All oligodeoxynucleotides were synthesized by Integrated DNA technologies (Integrated DNA technologies (IDT), Belgium) in PAGE grade.

### Acoustophoretic microfluidic chip design

The acoustophoretic microfluidic chip was fabricated in a 300 μm thick 3 inch silicon wafer using conventional photolithography and wet etching protocols^33^. In brief, wet etching of <100> silicon in KOH (220 g KOH in 550 ml MilliQ H_2_O) resulted in microfluidic-channels followed by dicing and anodic bonding to a 1.1 mm thick borosilicate glass lid. The width of the pre-focusing channel was 300 μm to match a wavelength resonance of 5 MHz, and the width for the main separation channel was 375 μm to match a half wavelength resonance of 2 MHz. Two piezoelectric transducing plates (Pz26, Ferroperm Piezoceramics AS, Denmark) were attached to the silicon chip using cyanoacrylate glue (Fig. 1). And lastly, Silicon tubing with an inner diameter of 1/100 inch was connected to microfluidic chip.

### Acoustophoresis set up

The glass syringes (Hamilton Bonaduz AG, Bonaduz, Switzerland) were connected to the inlet and the outlet of the side channels of the acoustophoresis chip. The center outlet was kept open and separated beads were collected through the center outlet. The syringe pumps (neMESYS; Cetoni GmbH, Korbussen, Germany) controlled flow rates of each syringe. The ultrasonic standing waves were generated and transferred (Agilent 33220A, Hewlett-Packard, Palo Alto, CA), to the two piezoelectric transducers. The function generators were connected to an amplifier for the pre-focus (Amplifier Research 75A250, Southerton, PA) and the main separation transducer (in-house build amplifier based on an LT1012 power amplifier (Linear Technology Corp., Milpitas, CA, USA)) respectively. The resonance frequency was 5 and 2 MHz for the pre-focusing and the main separation transducer, respectively. The temperature was controlled to 25 °C using a Peltier-controller (TC2812; Cooltronic GmbH, Beinwil am See, Switzerland) and the applied voltage was monitored using an oscilloscope (TDS 2120; Tektronix).

### Immobilization of the target protein on magnetic beads

Target protein PSA was immobilized on p-toluenesulfonyl (tosyl)-activated magnetic beads, 2.8 μm diameter, (Dynabeads M-280, Invitrogen Dynal, USA). The tosyl groups on the magnetic beads react with primary amino groups of the protein. Briefly, 20 μg PSA dissolved in 230 μl phosphate-buffered saline (PBS) buffer was added to washed magnetic beads (5 mg). This bead-protein mixture was incubated for 24 h at 37 °C with mild rotation and the beads were blocked with 0.5% BSA for additional 1 hour according to the manufacturer’s protocol. After the coating and blocking procedure, the amount of bound PSA on the beads was calculated through measuring the amount of unbound PSA by microBCA test (Pierce, Rockland, IL, USA), and target immobilized beads were stored at 4 °C until use.

### Screening of aptamer using acoustophoresis

A random oligonucleotide library of 1.2 × 10^14^ single-stranded DNA (ssDNA) molecules containing a central random 40-nucleotide region flanked on both sides by known 18-nucleotide primer binding regions was used.

Binding reactions were performed in binding buffer (2 mM MgCl_2_, and 0.02% Tween-20 in PBS, pH 7.4). Before the binding step, ssDNAs were treated by heating at 95 °C for 10 min and fast cooling at 4 °C for 15 min followed by incubation at 25 °C for 8 min as a pre-requisite for stable secondary ssDNA structure. Then the SELEX process was initiated by first incubation of PSA coated beads with 1.2 × 10^14^ of random ssDNAs from the library in a total reaction volume of 500 μl of binding buffer for 30 min. The ssDNAs bound to the target were separated from unbound or weakly bound ssDNAs by washing with acoustophoretic separation. After collecting the separated beads, the bound ssDNAs were eluted by suspending the beads containing ssDNAs bound to PSA in 200 μl of elution buffer (40 mM Tris-HCl, 10 mM EDTA, 3.5 M urea, 0.02% Tween-20, pH 8.0). The elution process was performed by heating the suspension at 95 °C for 10 min with mild shaking of the beads bound to target oligonucleotides. The eluted PSA-bound ssDNA fraction was purified by ethanol precipitation and amplified by polymerase chain reaction (PCR) using a fluorescent forward primer and an unlabeled reverse primer.

A double-stranded DNA (dsDNA) product was obtained after PCR using the following program: initial denaturation for 5 min at 94 °C and 10 cycles for 1 min at 94 °C, 1 min at 53 °C, 1 min at 72 °C, and extension for 10 min at 72 °C. The PCR product was subjected to denaturing polyacrylamide gel electrophoresis to isolate ssDNA fractions containing potential aptamer candidates from the PCR product (Fig. 2). The fluorescent ssDNA fractions were excised from gel and eluted using elution buffer (500 mM ammonium acetate, 0.1 mM EDTA, 0.1% sodium dodecyl sulfate) by crushing the gel; DNA was purified using ethanol precipitation. Purified ssDNA was re-dissolved in 500 μl of binding buffer and applied to the second selection round, and the entire process was repeated for the subsequent selection process to enrich the aptamers until the binding ratio (bound DNA/inlet DNA × 100) was saturated.

In addition, to avoid non-specific binding, the aptamer pool was subjected to counter selection steps against bare magnetic beads and counter targets including HSA, fibrinogen and IgG, which are known to be the most 3 abundant proteins in blood. For counter selection, the ssDNA pool from the 3^rd^ round of enrichment was incubated with bare beads first. The ssDNA pool that did not bind to the bare beads was separated using magnetic separation and then subjected to the mixture of PSA coated beads and counter targets including HSA, IgG and fibrinogen, which are freeform in the buffer condition. Counter selection was conducted after the 3^rd^ round until enrichment was finished.

### Next Generation Sequencing

NGS was performed using Illumina’s Mi-seq (Illumina, USA). The selected pools were PCR amplified to dsDNAs with an optimum cycle by primers (forward, reverse primer with 18mer size), and then purified by MinElute Kit (Qiagen, Germany). The purified dsDNAs were amplified using sequencing primers (with 6 bp barcodes) by PCR and purified before sequencing. For quality control purposes, we filtered out sequences of incorrect length. The number of unique sequences in the randomized 40 nucleotides was computationally counted after trimming the primer-binding regions for downstream analysis.

### Characterization of the aptamer candidate with Biacore

PSA and three counter proteins were immobilized on a CM5 sensor chip (Biacore AB, Uppsala, Sweden) to evaluate the binding characteristics of the aptamer candidates. Amine coupling was performed at a flow rate of 5 μl/min in HBS-EP (GE healthcare, USA). The CM5 chip surface was activated for 10 min using 0.2 M EDC/0.05 M NHS. After the injection of 10 μg/ml protein in 10 mM sodium acetate (pH 4.5), 5 μl of 1 M ethanolamine (pH 8.0) was used for blocking the remaining activated groups. Binding analysis was performed at a flow rate of 15 μl/min with PBSM (PBS pH 7.4, 2 mM MgCl_2_) at 25 °C. Each of the aptamer candidates were dissolved in PBSM and 30 μl of 100 nM ssDNA samples were injected for kinetic analysis with 2 min dissociation time. To evaluate dose dependent affinity of aptamer to target, two-fold serial dilutions of the aptamer samples were tested (from 3 nM to 50 nM of AS2).

For the specificity test, 100 nM of each aptamer candidate was injected to the CM5 chip treated with each protein of 10 μg/ml and ethanolamine blocking.

After each sample injection, the chip was regenerated by 5 μl of 0.5 M ethanolamine and extra washing. A blank flow cell (fc1) was used as a reference cell to correct the bulk refractive index by subtracting the response signal of the reference cell from the signals of protein immobilized cells. The dissociation constants of the aptamers were calculated by using BIAevaluation software (version 4.1, Biacore). For the kinetic analysis, the binding curve of the SPR signal was plotted in a Michaelis-Menten equation of  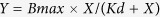 and the R^2^ value was calculated to confirm the fitting goodness. Bmax is the maximum specific binding in the response units and K_d_ is the equilibrium binding constant, which is the aptamer’s concentration required to achieve a half-maximum binding at equilibrium.

## Additional Information

**How to cite this article**: Park, J.-W. *et al.* Acousto-microfluidics for screening of ssDNA aptamer. *Sci. Rep.* **6**, 27121; doi: 10.1038/srep27121 (2016).

## Figures and Tables

**Table 1:** A comparison of various types of PSA binding molecules’ dissociation constant.

Dissociation constant	Type	Sequence	References
100 nM>	DNA	tttttAATTAAAGCTCGCCATCAAATAGCttt	56
65 nM	Peptide		57
630 nM	2F-RNA	2′F-AGCUCCAGAAGAUAAAUUACAGGUACGGUUCACGCCUGUCUCAUGCUGACUAAGAAAGUUUAGCAACUAGGAUACUAUGACCC	58
–	RNA	CCGUCAGGUCACGGCAGCGAAGCUCUAGGCGCGGCCAGUUGC	59
0.7 nM	DNA	GGGCGGGGCGGACGAGACAGTAAGGGCTGTGGGTGTGGTG	This Research
0.3 nM	antibody		48
